# Exposure to Di-(2-ethylhexyl) Phthalate During Perinatal Period Gender-Specifically Impairs the Dendritic Growth of Pyramidal Neurons in Rat Offspring

**DOI:** 10.3389/fnins.2018.00444

**Published:** 2018-07-24

**Authors:** Mingdan You, Jing Dong, Yuanyuan Fu, Zhangzhao Cong, Hui Fu, Lingling Wei, Yi Wang, Yuan Wang, Jie Chen

**Affiliations:** Department of Occupational and Environmental Health, School of Public Health, China Medical University, Shenyang, China

**Keywords:** DEHP, neurotoxicity, endocrine-disrupting chemicals, hippocampus, dendrite

## Abstract

Di-(2-ethylhexyl) phthalate (DEHP), as a prevalent xenoestrogen endocrine disrupter, is omnipresent in the environment and commonly used in polyethylene plastic products. Although DEHP has potential adverse effects on multisystem organs, damage to the central nervous system is more significant. However, the consequences and mechanisms of DEHP exposure remain to be explored. The aim of this study was to investigate the effects and related mechanisms of maternal DEHP exposure on dendritic development of hippocampal pyramidal neurons in a rat model. Pregnant Wistar rats were intragastrically administrated either vehicle or DEHP (30, 300, and 750 mg/kg/d) from gestation day 0 to postnatal day (PN) 21. The dendritic length and complexity of dendritic arbors’ pattern in pyramidal neurons of the hippocampus were measured using Golgi–Cox staining and Sholl analysis. The expression of dendritic development-related proteins was detected using western blot and immunofluorescence staining. DEHP-treated male but not female pups showed an obvious decrease in the total length and branching numbers of basal dendrites on PN7, PN14, and PN21. The phosphorylation of MAP2c, stathmin, and JNK1 in the male pup hippocampus was significantly decreased in DEHP treatment groups compared to controls. However, protein expression alteration in the hippocampus of female offspring was not observed. In summary, our study indicated that DEHP has a gender-specific negative impact on the dendritic growth of CA1 pyramidal neurons in male offspring of a rat model of DEHP exposure. The adverse impact may be related to the dysregulation of phosphorylated and total MAP2c and stathmin mediated by JNK1.

## Introduction

Phthalates (PAEs) are common plasticizers used to produce the required flexibility and plasticity in polyvinylchloride (PVC), which is ubiquitously used in the manufacture of consumer goods, including food containers, children’s toys, pharmaceuticals, nutritional supplements, and medical devices (Haishima et al., 2005; Shelby, 2006; Rudel et al., 2011; Kelley et al., 2012). Because PAEs are not chemically covalently bound in the polymer, they can be easily separated out from PVC consumer products to persist in the environment. A high concentration of PAEs in the environment has been detected in several places around the world, including at a concentration up to 63,000 μg/kg in the agricultural soils of Spain (Gao and Wen, 2016). Environmental PAEs directly or indirectly enter the human body to adversely impact human health (Sui et al., 2014). As environmental endocrine-disrupting chemicals, PAEs exert pleiotropic deleterious effects on the immune, reproductive, cardiovascular, and nervous systems (Heudorf et al., 2007; Zarean et al., 2016; Wang et al., 2017).

Di-(2-ethylhexyl) phthalate (DEHP), which accounts for 50% of total PAE production, is a global environmental pollutant (Guo and Kannan, 2012). DEHP can aggregate in the placenta and mother’s milk, cross the placental and blood–brain barrier, and contribute to bioaccumulation in the fetus and infants during gestational and lactational periods (Stroheker et al., 2006; Grandjean and Landrigan, 2014). Furthermore, infants and children are more vulnerable to environmental pollutants, especially during the rapid physical development stage of their key organs because of their higher metabolic rate (Yaghjyan et al., 2015; Arbuckle et al., 2016). DEHP exposure causes severe health hazards for infants and children. The mechanisms involving reproductive damage by DEHP in infants and children have been systematically studied (Lyche et al., 2009; Martino-Andrade and Chahoud, 2010; Niermann et al., 2015). It is worth noting that nervous system damage in infants is likely to be permanent when it occurs during the perinatal period, a crucial window of development (Rice and Barone, 2000). Moreover, many epidemiological studies have confirmed that DEHP exposure is positively associated with the pathogenesis of neurodevelopmental disorders, such as autism spectrum disorder and attention deficit hyperactivity disorder symptoms (Benson, 2009; Pan et al., 2011; Keune et al., 2012; Upson et al., 2013; Chopra et al., 2014; Kim et al., 2015; Park et al., 2015; Philippat et al., 2015). Therefore, the negative effects of perinatal exposure to DEHP on the neurodevelopment of offspring have aroused widespread concern (Pan et al., 2011; Philippat et al., 2015; Zarean et al., 2016). However, only a few experimental studies have illustrated the effects of DEHP on neurodevelopmental disorders (Wang et al., 2014; Dai et al., 2015) and the potential mechanisms remain unclear.

The hippocampus, a major component of the brain of humans and other mammals that is located bilaterally in the medial temporal lobe and underneath the cortical surface, plays critical roles in learning and memory (Schlichting et al., 2014). As the main neurons in the hippocampus, pyramidal neurons of the CA1 subregion receive converging inputs from multiple input pathways, which is an indispensable part of the neural signal transduction pathway in the hippocampus (Kesner et al., 2004). Additionally, the establishment of precise neural circuits during development depends on the normal development of dendrites (Goodman and Shatz, 1993). Dendritic tree size and complexity can modulate the propagation of action potentials (Vetter et al., 2001), influence the firing pattern of a neuron, and determine the number of synaptic connections (Mainen and Sejnowski, 1996; Hausser et al., 2000). Dendrites appearing shorter as well as less intricate dendritic arborization may contribute to neurological disease (Lin and Koleske, 2010; de Anda et al., 2012; Park et al., 2015).

The cytoskeleton is an important component of dendrite formation and plays a leading role in regulating neuronal morphology. Microtubules (MTs) are the backbone of the cytoskeleton (Maccioni and Cambiazo, 1995). Further, as important components closely related to intracellular motion, the fixation of cell organelles, the maintenance of cell morphology, cell migration, signal transduction, and cell division, MTs are imperative for the complex morphology of neurons and the formation of precise neural circuits (Vega and Solomon, 1997; Sanchez et al., 2000). Research has found that MTs are regulated by structural MT-binding proteins including severing proteins, catastrophe factors (e.g., stathmin), and MT assembly-promoting proteins referred to as MT-associated proteins (MAPs) (Sanchez et al., 2000). MAP2 is abundant in the mammalian central nervous system (CNS) and is predominantly expressed in neurons; its phosphorylation seems to control its association with the cytoskeleton (Brugg and Matus, 1991). In contrast, stathmin is a MT-destabilizing factor that is highly expressed in the developing nervous system. Stathmin phosphorylation can suppress MT-destabilizing activities (Di Paolo et al., 1997). The physiological activities of MAP2 and stathmin are regulated by c-Jun N-terminal kinase 1 (JNK1), which is necessary for maintaining the cytoskeletal integrity of dendrites in the CNS (Chang et al., 2003; Bogoyevitch and Kobe, 2006; Yeap et al., 2010).

Although neurodevelopmental impairment in children caused by environmental DEHP exposure during pregnancy has attracted extensive concern, the specific damaging effects and the potential mechanisms involved remain to be clarified (Kim et al., 2009; Yen et al., 2011; Chopra et al., 2014).

## Materials and Methods

### Animals and Treatment

Adult female Wistar rats were obtained from the Center for Experimental Animals at China Medical University (Shenyang, China) with a National Animal Use License number of SYXK-LN2013-0007. All experiments and surgical procedures were approved by the Animal Use and Care Committee at China Medical University, which complies with the National Institutes of Health Guide for the Care and Use of Laboratory Animals. All efforts were made to minimize the number of animals used and their suffering. Rats were housed at a temperature of 24 ± 1°C with 12 h light/12 h dark cycles and humidity 50–60%. Food and water were provided *ad libitum*. Animals were housed for 1 week before being entered into the study. The female rats were randomly assigned among the treatment groups and then mated with normal male rats (♀/♂ = 1:2). The day of the vaginal plug was taken as gestation day 0 (GD0), and the pregnant dams were each placed in an individual cage. A total number of 20 dams per dose were used. All the dams were allowed to feed their offspring until weaning on postnatal day (PN) 21.

Pregnant females were administered intragastrically vehicle (corn oil) or DEHP (sigma, United States) in vehicle at a dose of 0, 30, 300, or 750 mg/kg/day by daily gavage from GD0 to PN21, with the doses corresponding to groups named hereafter as control group, 30 mg/kg/d group, 300 mg/kg/d group, and 750 mg/kg/d group, respectively. The intragastric administration was performed at a fixed time every day, at 8:30–9:30 a.m. The exposure dose of dams was calculated by a conversion formula and chosen as 30 mg/kg/d (Campioli et al., 2014). The dosage of 300 mg/kg/d converted to human equivalent dose based on the body surface area was corresponded to the no-observed-adverse-effect level (NOAEL, 48 mg/kg/d) of DEHP in humans (Reagan-Shaw et al., 2008). The dosage of 750 mg/kg/d is known to be able to induce adverse impact in rats without causing systemic toxicity (Shelby, 2006).

### Golgi–Cox Staining

The staining procedures were performed according to the manufacturer’s instructions (Hitobiotec, Inc., United States). On PN7, PN14, and PN21, six pups (gender balance) in each group were anesthetized, and their brains were removed. The brains were rinsed briefly in double distilled water and immersed in the impregnation solution at room temperature in the dark. The impregnation solution was refreshed on the next day and kept for 2 weeks to reduce background staining. Next, the tissues were transferred into solution C at 4°C in the dark and refreshed the next day, and then kept for another 2–3 days. Afterward, the brains were cut in 80–120 μm thick coronal slices using a vibratome (VT1200S, Leica Biosystems Nussloch GmbH). Brains were sectioned in a serial manner when the intact structure of the hippocampus was observed in the slices. Sections were collected onto SuperFrost Plus slides and allowed to dry naturally at room temperature. The dried sections were rinsed with deionized water, dehydrated through a graded series of ethanol, cleared in 100% xylene, and covered slipped using a resinous medium. The dendritic branches rooting from six intact neurons per animal were traced to analyze the morphogenesis of the dendritic branch. All traced neurons were from the same anatomical position in the CA1 subregion of the dorsal hippocampus. ImageJ software was used to quantify the dendritic tracings. The total length of the basal or apical dendrites in pyramidal neurons was a statistical parameter. The dendritic branching complexity was another evaluation parameter, which counted the numbers of intersections between the dendrites and an overlaid concentric sphere at 10 μm intervals using Sholl’s method (Sholl, 1953).

### Western Blot

On PN7, PN14, and PN21, the hippocampus tissues of four males and four females were taken from different litters from each group and respectively homogenized with the help of an ultrasonic cell disrupter in cold RIPA buffer with protease and phosphatase inhibitors. The protein concentration was measured using a BCA Kit (Thermo Scientific, Rockford, IL, United States). The process of western blot refers to previous studies (Wang et al., 2017). In short, after electrophoresis, transferring to the PVDF membrane, and blocking with skim milk, membranes were incubated overnight at 4°C with primary rabbit anti-phospho-MAP2 (Ser136) (#4541, Cell Signaling Technology; 1:500), rabbit antibody anti-MAP2 (#8707, Cell Signaling Technology; 1:500), rabbit antibody anti-phospho-stathmin (Ser38) (#4191, Cell Signaling Technology, 1:500), rabbit antibody anti-stathmin (ab2906, Abcam, 1:5000), rabbit antibody anti-JNK1 (ab110724, Abcam, 1:500), rabbit antibody anti-phospho-JNK1 (Thr183) (ab47337, Abcam, 1:500), and rabbit antibody anti-β-actin (#4970, Cell Signaling Technology; 1:500). After three rinses, the membranes were incubated with horseradish peroxidase-conjugated secondary antibody (ZB-2301, Zhongshan Biotechnology; goat anti-rabbit, 1:3000 dilution) for 1 h at room temperature and were rinsed again similarly. A bioanalytical imaging system (Azure Biosystems, Inc.) was used to visualize the specific protein. The relative density was quantified by using ImageJ software.

### Immunofluorescence

On PN7, PN14, and PN21, a total of eight pups (gender balance) from each group were perfused transcardially with normal saline, followed by 4% paraformaldehyde in 0.1 M potassium phosphate buffer (pH = 7.4). Following fixation, intact brains were quickly removed and submerged into 4% paraformaldehyde overnight. After dehydration with ethanol of gradient concentration, xylene transparency, and paraffin embedding, coronal sections were sectioned at 4 μm thickness by a rotary microtome (RM2255, Leica Biosystems Nussloch GmbH). Brains were sectioned in a serial manner when the intact structure of the hippocampus was observed in the slices. The sections were deparaffinized by xylene for 10 min followed by 100% ethanol and then were rinsed three times in phosphate buffered saline (PBS). After blocking for 30 min at room temperature with 10% goat serum and incubation at 4°C overnight in a primary rabbit anti-phospho-MAP2 (Ser136) antibody (#4541, Cell Signaling Technology, 1:100) or the rabbit anti-phospho-stathmin (Ser38) antibody (#4191, Cell Signaling Technology, 1:100), sections were washed and incubated with fluorescently labeled secondary antibodies TRITC (ZF-0316, Zhongshan Biotechnology, Beijing, China, 1:100) at room temperature for 2 h in a dark place, followed by washing in PBS. Next, the nuclei were stained by DAPI (D3571, Thermo Fisher Scientific). The pictures were obtained from fluorescence microscopy (BX61+DP-71, Olympus/IPP, JAPAN/United States) under the same conditions at a magnification of 200× (objective 20× and ocular 10×). We selected three different fields from each of the three slices and calculated the average values. The fields of analysis are from the CA1 subregion of the dorsal hippocampus. The representative images in the figure were taken from the same fields. Eight rats from each group were used to obtain an overall mean value for subsequent statistical analysis for each time point.

### Statistical Analysis

All analyses were carried out using SPSS software, version 21.0 (SPSS, Inc., Chicago, IL, United States). All experiments were performed at least triplicate, and data were presented as the mean ± standard deviation (SD). A one-way analysis of variance followed by the Student–Newman–Keuls test was used to compare the treated groups with control group. *p* < 0.05 was considered statistically significant.

## Results

### Maternal Exposure to DEHP Caused No Significant Changes to Bodyweight and Hippocampal Weight of Pups on PN7, PN14, and PN21

The bodyweight and the hippocampal weight of pups were measured to estimate the effects on the physical and hippocampal developments of offspring following maternal exposure to DEHP. For all offspring, significant changes in bodyweight and hippocampal weight in all groups at all time points were not observed. These results showed that maternal exposure to DEHP does not cause systemic damage to the body and the hippocampus of offspring (**Supplementary Figure S1**).

### Maternal Exposure to DEHP Impaired the Dendritic Morphology of Pyramidal Neurons From Rat Offspring

**FIGURE 1:**
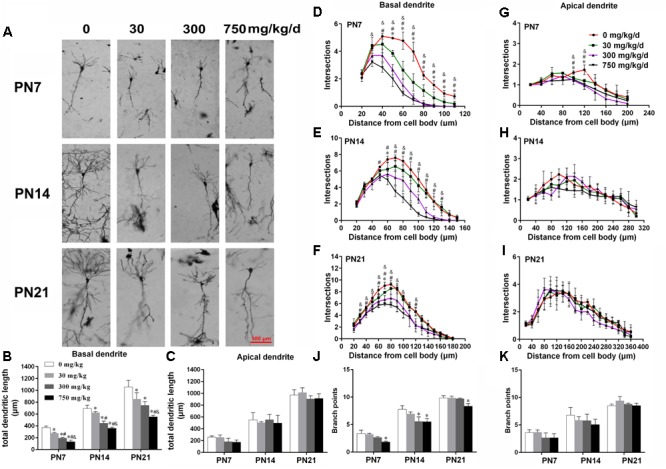
Maternal exposure to DEHP has a negative effect on the development of pyramidal neurons in the hippocampal CA1 of male pups. **(A)** Graphs are representative tracings of pyramidal neuron dendrites of the hippocampal CA1 region in male pups from DEHP-exposed rats (0, 30, 300, and 750 mg/kg) at PN7, PN14, and PN21, respectively. Scale bar = 100 μm. **(B,C)** Bar graphs display the total basal and apical dendrite length of male pups, respectively, at all time points. **(J,K)** Bar graphs display the total number of branching points of the main basal and apical dendrites of male pups, respectively, at all time points. Values of bar graphs are expressed as mean ± standard deviation (SD). ^∗^*p* < 0.05 vs. control; ^#^*p* < 0.05 vs. 30 mg/kg/d; ^&^*p* < 0.05 vs. 300 mg/kg/d. The number of dendritic intersections in CA1 pyramidal neuron between the dendrites and the soma was measured using Sholl analysis on PN7, PN14, and PN21 **(D–I)**. **(D–I)** Graphs represent the number of dendritic intersections in basal and apical dendrites of male pups. Values are shown as mean ± SD. ^∗^*p* < 0.05 for 30 mg/kg/d vs. control groups; ^#^*p* < 0.05 for 300 mg/kg/d vs. control groups; ^&^*p* < 0.05 for 750 mg/kg/d vs. control groups.

**FIGURE 2:**
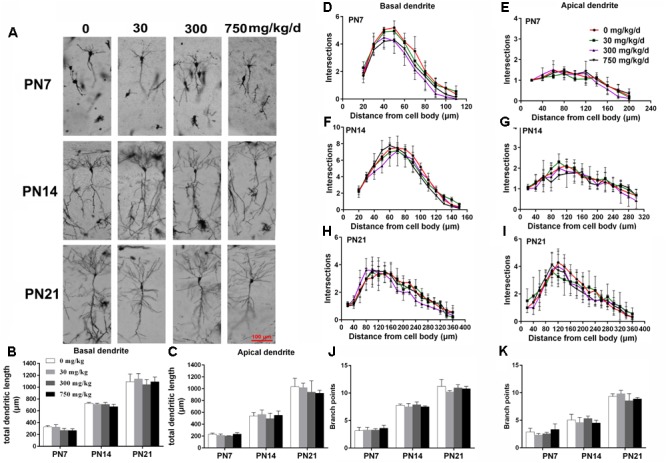
Maternal exposure to DEHP has no effect on the development of pyramidal neurons in the hippocampal CA1 of female pups. **(A)** Graphs are representative tracings of pyramidal neuron dendrites of the hippocampal CA1 region in female pups from DEHP-exposed rats (0, 30, 300, and 750 mg/kg) at PN7, PN14, and PN21, respectively. Scale bar = 100 μm. **(B,C)** Bar graphs display the total basal and apical dendrite length of female pups, respectively, at all time points. **(J,K)** Bar graphs display the total number of branching points of the main basal and apical dendrites of female pups, respectively, at all time points. Values of bar graphs are expressed as mean ± standard deviation (SD). ^∗^*p* < 0.05 vs. control; ^#^*p* < 0.05 vs. 30 mg/kg/d; ^&^*p* < 0.05 vs. 300 mg/kg/d. The number of dendritic intersections in CA1 pyramidal neuron between dendrites and soma was measured using Sholl analysis on PN7, PN14, and PN21 **(D–I)**. **(D–I)** Graphs represent the number of dendritic intersections in basal and apical dendrites of female pups. Values are shown as mean ± SD. ^∗^*p* < 0.05 for 30 mg/kg/d vs. and control groups; ^#^*p* < 0.05 for 300 mg/kg/d vs. control groups; ^&^*p* < 0.05 for 750 mg/kg/d vs. control groups.

The development of dendrites is vital for proper neuronal connectivity and networking in the brain (Valnegri et al., 2015). The total length, total branching number, and complexity of the dendritic branching pattern of apical and basal dendrites were measured to estimate the impairment of the development of CA1 pyramidal neurons of offspring following maternal exposure to DEHP (**Figures 1, 2**). Maternal DEHP exposure produced a marked decrease in the total lengths of basal dendrites in treated male pups, but not in treated female pups (**Figures 1B, 2B**). For DEHP-treatment male pups, compared with the control group, the total lengths of basal dendrites (**Figure 1B**) were reduced by 26.31, 9.99, and 19.28% on PN7, PN14, and PN21 in the 30 mg/kg/d group, respectively (*p* < 0.05). The total lengths of basal dendrites in 300 and 750 mg/kg/d groups displayed a more remarkable decrease than those of the 30 mg/kg/d group compared with the control group on PN7, PN14, and PN21, respectively (*p* < 0.05). As for the total lengths of apical dendrites, a significant difference in male pups and female pups in all groups at all time points was not observed (**Figures 1C, 2C**).

The complexity of the dendritic branching pattern was also measured and analyzed. The intersection numbers in the basal dendrites of the DEHP-exposed male offspring were significantly reduced relative to those in the control group on PN7, PN14, and PN21 (**Figures 1D–F**; *p* < 0.05). However, for apical dendrites, the intersection numbers in DEHP-exposed male offspring slightly declined in a significant manner on PN7 compared with those of the control group (**Figure 1G**; *p* < 0.05). For female offspring, significant changes in the intersection numbers of basal and apical dendrites in all groups at all time points were not observed (**Figures 2D–F**).

The total number of branching points of the basal and apical dendrites were also counted. For male pups, in the 750 mg/kg/day group, significant decreases of the branching points in basal dendrites were observed relative to the control group on PN7, PN14, and PN21 (**Figure 1J**; *p* < 0.05). In addition, the branching points of basal dendrites in pups of the 300 mg/kg/day group were significantly reduced compared with those of the control group on PN14 (**Figure 1J**; *p* < 0.05). However, for apical dendrites, significant changes of the branching points in DEHP-exposed male offspring were not observed at all time points compared with those of the control group (**Figure 1K**). For female pups, significant alterations of the number of branching points in the basal and apical dendrites in the DEHP treatment groups compared to control groups at all time points were not observed (**Figures 2J,K**). These results showed that maternal exposure to DEHP has a negative effect on the development of pyramidal neurons in the hippocampal CA1 of male pups.

### Maternal Exposure to DEHP Reduced MAP2 in the Hippocampus of Male Pups

**FIGURE 3:**
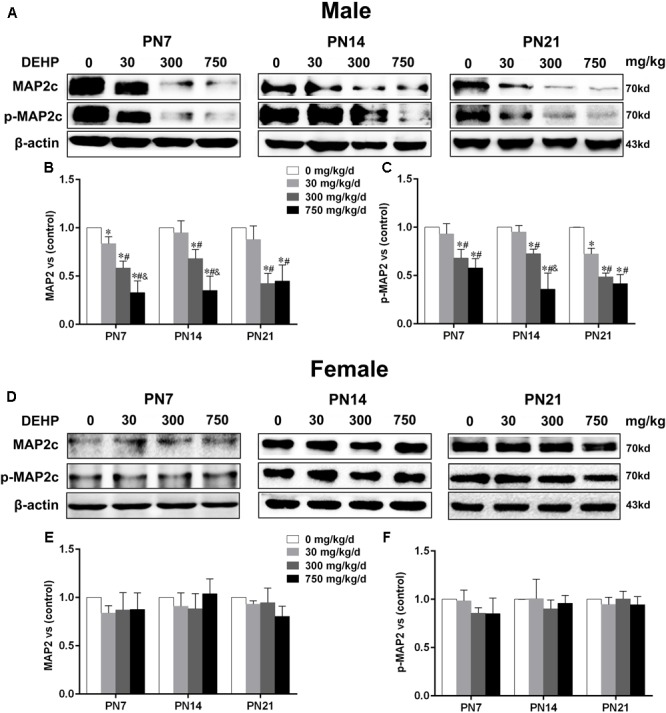
Maternal exposure to DEHP reduced MAP2c and phospho-MAP2c in male pups and without significant changes in female pups on PN7, PN14, and PN21. The upper bands, in western blots, show representative findings for male **(A)** and female pups **(D)** after corn oil (control) or DEHP treatments on PN7, PN14, and PN21. The lower bar graphs display the results of the semiquantitative measurement of MAP2c and p-MAP2c in male **(B,C)** and female pups **(E,F)**. Values are expressed as mean ± SD (*n* = 4 pups from 4 L). The mean expression of protein in pups from 30, 300, and 750 mg/kg/d groups is shown as the fold-change compared with the mean expression in the control group that has been ascribed an arbitrary value of 1. ^∗^*p* < 0.05 vs. 0 mg/kg/d; ^#^*p* < 0.05 vs. 30 mg/kg/d; ^&^*p* < 0.05 vs. 300 mg/kg/d.

MTs are essential for dendritic growth and the maintenance of dendritic morphology. MAP2, as an MT-stabilizing protein, maintains the integrity of the MTs of neurons and plays an important role in the development of dendrites (Harada et al., 2002). The expressions of MAP2c and phospho-MAP2c in the hippocampus of pups were detected by using the western blot (**Figures 3A,D**). For male pups, compared with the control group, MAP2c expression and phospho-MAP2c were significantly downregulated by DEHP exposure of 300 and 750 mg/kg/day on PN7, PN14, and PN21 (**Figures 3B,C**; p < 0.05). In addition, the total MAP2c protein levels in pups of the 30 mg/kg/day group were significantly reduced compared with those of the control group on PN7 (p < 0.05). We also found significantly decreased phospho-MAP2c levels on PN21 in pups of the 30 mg/kg/day group (p < 0.05). For females, the expressions of MAP2c and phospho-MAP2c were not significantly changed in all treatment groups at all time points (**Figures 3E,F**).

**FIGURE 4:**
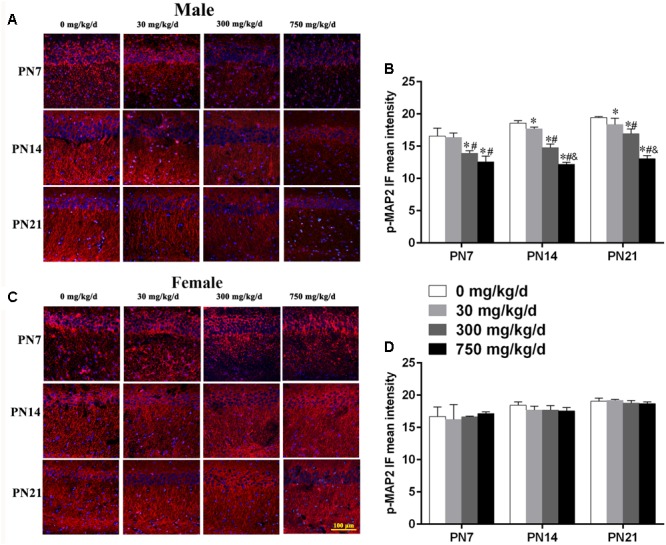
Maternal exposure to DEHP decreased phospho-MAP2c in the CA1 region in male pups and without significant changes in female pups on PN7, PN14, and PN21. **(A,C)** Representative immunofluorescence (IF) photomicrographs that show the expression of phospho-MAP2c (*red*) and DAPI (*blue*) at PN7, PN14, and PN21 in the CA1 subfield. The representative images in the figure were taken from the same fields. The images were obtained under the same conditions at a magnification of 200×. Scale bar = 100 μm. **(B,D)** Bar graphs display the results of the mean intensity of phospho-MAP2 immunofluorescence of all groups at all time points. Values are expressed as mean ± SD (*n* = 4 pups from 4 L). ^∗^*p* < 0.05 vs. 0 mg/kg/d; ^#^*p* < 0.05 vs. 30 mg/kg/d; ^&^*p* < 0.05 vs. 300 mg/kg/d.

Furthermore, to further determine the activation of MAP2c, the level of phospho-MAP2c in the hippocampal CA1 region of pups was also detected using immunofluorescence staining (**Figures 4A,C**). For males, positive staining of phospho-MAP2c was observed to be significantly reduced in the 30, 300, and 750 mg/kg/day groups compared with the control group (**Figure 4B**; p < 0.05). MAP2c phosphorylation in pups from the 30 mg/kg/day group was significantly reduced compared with that of pups from the control group on PN14 and PN21 (p < 0.05), while a significant alteration was not found on PN7 (**Figure 4B**). In terms of female pups, a significant change in phospho-MAP2c in DEHP-exposed groups at all time points was not found (**Figure 4D**). The above results indicated that DEHP exposure decreased the expression of phospho-MAP2c in the hippocampal CA1 subregion in a sex-specific manner.

### Maternal Exposure to DEHP Enhanced Stathmin Expression in the Hippocampus of Male Pups

**FIGURE 5:**
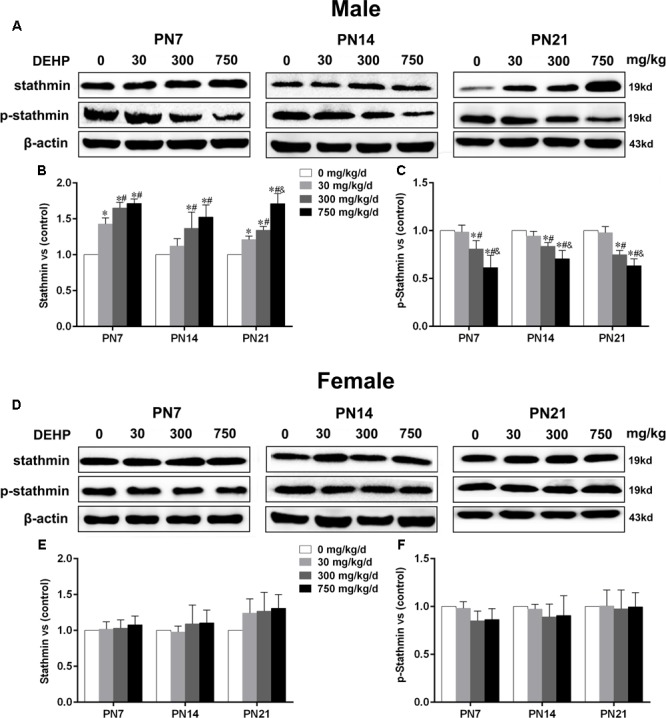
Maternal exposure to DEHP enhanced the levels of stathmin and decreased phospho-stathmin in male pups and without significant changes in female pups on PN7, PN14, and PN21. The upper bands, in western blots, show representative findings for male **(A)** and female pups **(D)** after corn oil (control) or DEHP treatments on PN7, PN14, and PN21. The lower bar graphs display the results of the semiquantitative measurement of stathmin and p-stathmin in male **(B,C)** and female pups **(E,F)**. Values are expressed as mean ± SD (*n* = 4 pups from 4 L). The mean expression of protein in pups from 30, 300, and 750 mg/kg/d groups is shown as the fold-change compared with the mean expression in the control group that has been ascribed an arbitrary value of 1. ^∗^*p* < 0.05 vs. 0 mg/kg/d; ^#^*p* < 0.05 vs. 30 mg/kg/d; ^&^*p* < 0.05 vs. 300 mg/kg/d.

Stathmin, an MT-destabilizing factor, is implicated in MT dynamics by modulating the formation and disassembly of MTs (Shumyatsky et al., 2005). Immunoblot analysis (**Figures 5A,D**) was conducted to investigate the effects of DEHP exposure on stathmin expression in the hippocampus. For male pups, in the 300 and 750 mg/kg/day groups, a significant downregulation of phospho-stathmin and upregulation of stathmin were observed relative to the control group on PN7, PN14, and PN21 (**Figures 5B,C**; p < 0.05). Moreover, compared with the control group, a significant increase in stathmin expression in the 30 mg/kg/day group on PN7 and PN21 was observed (**Figure 5B**; p < 0.05), while no obvious alteration was observed on PN14. Furthermore, the 30 mg/kg/day group showed slightly decreased phospho-stathmin levels on PN7, PN14, and PN21, but there was no significant difference. For female pups, significant changes in stathmin and phospho-stathmin among DEHP-exposed groups at all time points were not observed (**Figures 5E,F**).

**FIGURE 6:**
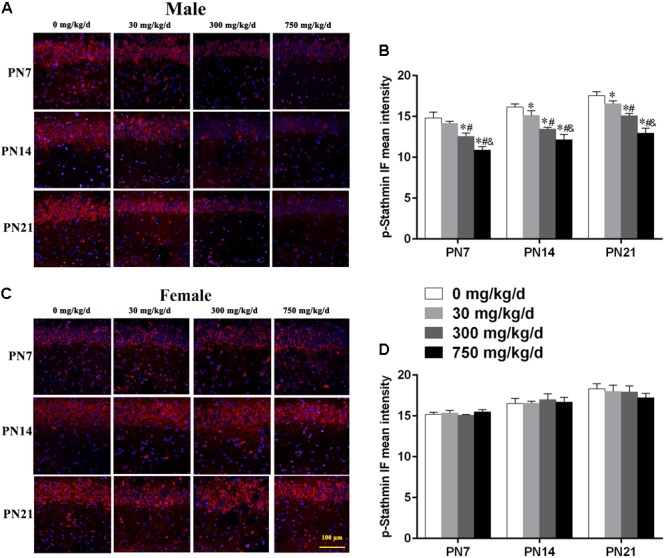
Maternal exposure to DEHP decreased phospho-stathmin in the CA1 region in male pups and without significant changes in female pups on PN7, PN14, and PN21. **(A,C)** Representative IF photomicrographs that show the expression of phospho-stathmin (*red*) and DAPI (*blue*) at PN7, PN14, and PN21 in the CA1 subfield. The representative images in the figure were taken from the same fields. The images were obtained under the same conditions at a magnification of 200×. Scale bar = 100 μm. **(B,D)** Bar graphs display the results of the mean intensity of phospho-MAP2 immunofluorescence of all groups at all time points. Values are expressed as mean ± SD (*n* = 4 pups from 4 L). ^∗^*p* < 0.05 vs. 0 mg/kg/d; ^#^*p* < 0.05 vs. 30 mg/kg/d; ^&^*p* < 0.05 vs. 300 mg/kg/d.

Similarly, we also conducted immunofluorescence staining (**Figures 6A,C**) to detect the expression of phospho-stathmin in the CA1 subfield. For male pups exposed to DEHP, a decreased mean intensity of phospho-stathmin positive staining in the CA1 subfield compared to the control group on PN7, PN14, and PN21 was observed (**Figure 6B**; p < 0.05). However, a significant difference between DEHP-exposed and control female pups was not found (**Figure 6D**). After the exposure of pups to DEHP, phospho-stathmin presented gender-specific abnormalities compared with the control group, with a decrease noted in male pups only.

### Maternal Exposure to DEHP Reduced Phospho-JNK1 Expression in the Hippocampus of Male Pups

**FIGURE 7:**
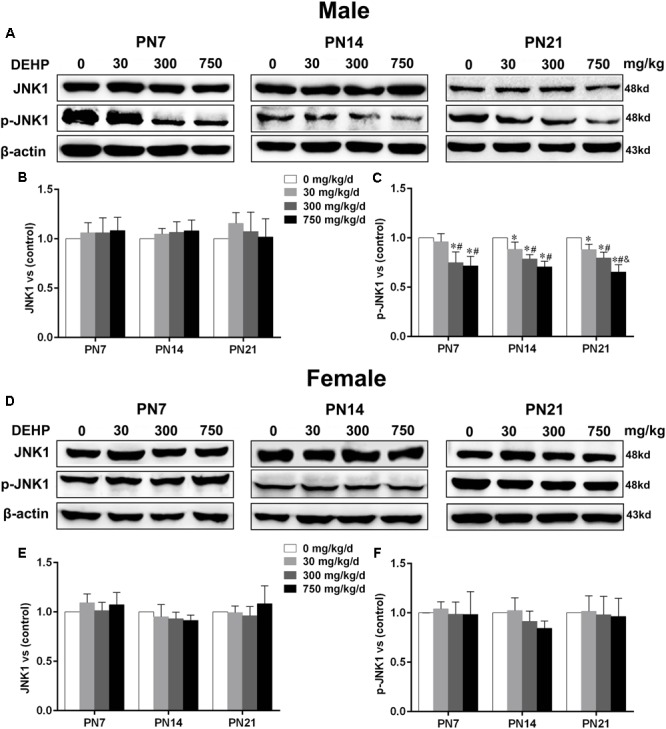
Maternal exposure to DEHP reduced JNK1 and phospho-JNK1 in male pups and without significant changes in female pups on PN7, PN14, and PN21. The upper bands, in western blots, show representative findings for male **(A)** and female pups **(D)** after corn oil (control) or DEHP treatments on PN7, PN14, and PN21. The lower bar graphs display the results of the semiquantitative measurement of JNK1 and p-JNK1 in male **(B,C)** and female pups **(E,F)**. Values are expressed as mean ± SD (*n* = 4 pups from 4 L). The mean expression of protein in pups from 30, 300, and 750 mg/kg/d groups is shown as the fold-change compared with the mean expression in the control group that has been ascribed an arbitrary value of 1. ^∗^*p* < 0.05 vs. 0 mg/kg/d; ^#^*p* < 0.05 vs. 30 mg/kg/d; ^&^*p* < 0.05 vs. 300 mg/kg/d.

Accumulating evidence suggested that JNK1 is directly involved in the regulation of MTs by controlling phosphorylation levels of MT-stabilizing/destabilizing factors, particularly phospho-MAP2 and phospho-stathmin (Chang et al., 2003; Yip et al., 2014). A statistically significant difference in expression of JNK1 in the hippocampus of DEHP-exposed male pups in comparison with the control group was not found (**Figures 7A,B**). A significant decrease was observed in the expression of phospho-JNK1 in the hippocampus of DEHP-exposed male pups compared with the control group (**Figures 7A,C**; *p* < 0.05). However, for female pups, significant alterations of JNK1 and phospho-JNK1 in the 30, 300, and 750 mg/kg/day groups compared to control groups at all time points were not observed (**Figures 7D–F**).

## Discussion

Di-(2-ethylhexyl) phthalate is omnipresent in the environment and can be bioaccumulated and concentrated by the food chain and water. Thus, a high concentration of DEHP can enter the human body and exert pleiotropic deleterious effects on health (Halden, 2010). Many epidemiological investigations have reported that maternal exposure to DEHP during the gestation period may lead to neural developmental anomalies of offspring (Chopra et al., 2014; Gascon et al., 2015; Verstraete et al., 2016). Additionally, many studies have shown that DEHP exposure to pregnant dams causes neurobehavioral disturbances in offspring, such as decreased spatial learning and memory, increased anxiety- and depression-like behaviors, and cognitive impairment (Sun et al., 2014; Dai et al., 2015; Xu et al., 2015). However, the underlying mechanism is unclear. Therefore, in the present study, we established an animal model by treating dams with DEHP (0, 30, 300, or 750 mg/kg/day) by intragastric administration to observe the effects of perinatal DEHP exposure on hippocampal dendritic development, and explored the underlying mechanisms involved.

Neuronal dendrites are important parameters in constructing accurate neural circuits (Libersat and Duch, 2004). Anomalous changes in the lengths and branching patterns of dendrites can greatly affect the computational power and performance of neurons and ultimately result in neurobehavioral abnormities (Bading et al., 2016). In the CA1 subregion, basal and apical dendrites are in different positions and have different structures that result in their specific innervational functions in hippocampal neural circuitry (Shinohara et al., 2012). In this study, we found that maternal exposure to DEHP decreased the length and branching complexity of basal dendrites in the hippocampal CA1 neurons of male pups, while alterations in apical dendrites were slight. In addition, several studies have shown that persistent organic pollutants as well as endocrine-disrupting chemicals may cause similar variations in the dendrites of hippocampal neurons (Hu et al., 2016; Kimura et al., 2016). However, another study reported that the total number of branches on the dendrites of CA1 neurons was not significantly different to controls following DEHP exposure from PN16 to 22 (Smith and Holahan, 2014). We speculate that DEHP administration time was one of the reasons for the results of the study not being consistent with our findings. It is generally accepted that the key developmental time period for pyramidal neurons in the hippocampal CA1 region is from birth to PN7 (Pokorny and Yamamoto, 1981). As the exposure time (PN16–22) that the study used was not within this key developmental time period, this may explain why detrimental effects on dendrites in the hippocampal CA1 region were not observed. Additionally, different doses and methods of exposure used in the study may also be the cause of inconsistency with our results. Thus, it is speculated that the atrophy of the dendrites after DEHP exposure during uterine and lactation contributed to the abnormal behavior of pups.

The formation of an intricate branch pattern of dendrites that depend on the stability of MTs is necessary to the integrity of the neural circuit. MAPs play major roles in promoting the assembly of tubulin, binding and stabilizing MTs, forming cross-bridge structures between MTs, and regulating kinesin- and dynein-dependent transport along MTs (Ma et al., 2014). MAP2, as a principal member of MAPs, is important for dendritic elongation *in vitro* and *in vivo* (Harada et al., 2002). Additionally, the phosphorylation of MAP2, which is involved in the polymerization of MTs, can regulate the development of the dendrites of neurons in the developmental stage (Diez-Guerra and Avila, 1993; Terabayashi et al., 2007). Therefore, MAP2 plays an important role in the stabilization of dendritic MTs and regulates the development of dendrites by changing the level of its phosphorylation in dendrites (Sanchez et al., 2000). In our study, we found that environmental exposure to DEHP decreased the expression of total MAP2c and phospho-MAP2c in the hippocampus of male pups on PN7, PN14, and PN21. Furthermore, immunofluorescence staining demonstrated the decreased expression of the phosphorylation MAP2c in the hippocampal CA1 subregion of the male. For dendritic development, phosphorylated and unphosphorylated versions of MAP2 are very important. It is speculated that the decrease in total MAP2c might contribute to the indirect decrease in phosphorylated MAP2c except for the direct reduction of activated MAP2c following DEHP exposure, and could ultimately lead to dendritic impairments. It is illustrated that DEHP exposure impairs the cytoskeleton proteins not only in normal function itself but also in the phosphorylation activation. Thus, the downregulation of total MAP2c and phospho-MAP2c may contribute to impairments of neuronal dendrites in the CA1 subregion following maternal exposure to DEHP.

Contrary to the effect of MAP2c, stathmin is an MT-destabilizing factor. Specifically, stathmin forms a ternary complex with two tubulin dimers that reduce the binding affinity of tubulin heterodimers, resulting in MT destabilization. However, it directly promotes MT catastrophic events through a direct interaction with MT ends (Gavet et al., 2002). Thus, stathmin overexpression can significantly limit dendritic arborization and reduce the dendritic growth during the development of the CNS. In this investigation, we showed that maternal exposure to DEHP significantly enhanced stathmin expression in the hippocampus on PN7, PN14, and PN21, respectively. It was speculated that maternal DEHP exposure may cause MT depolymerization in the hippocampus of pups by upregulating the expression of stathmin. Furthermore, stathmin phosphorylation was significantly reduced on PN7, PN14, and PN21 following the maternal DEHP treatment. As a ubiquitous cytosolic phosphoprotein located in the cytoplasm, stathmin is highly expressed in the brain and is phosphorylated by various protein kinases in response to extracellular signals (Sobel, 1991; Beretta et al., 1993). It has been clearly demonstrated that stathmin family proteins can be phosphorylated by JNK1 at the ser-38 site, that then directly decreases the ability of stathmin to inhibit MT polymerization and contributes to MT homeostasis and axondendritic growth during brain development (Di Paolo et al., 1997; Yip et al., 2014). Therefore, our study further confirmed that maternal exposure to DEHP inhibited the phosphorylation of stathmin. These results indicated that the upregulation of stathmin expression and the downregulation of phosphorylated stathmin caused by perinatal DEHP treatment could inhibit the development of pyramidal neuron dendrites in the hippocampal CA1 subregion by reducing MTs stability. Our study indicated that DEHP exposure causes impairments of the dendritic growth in pyramidal neurons, as well as the dysregulation of MAP2c and stathmin. MAP2 and stathmin are the substantial proteins in the brain. It is speculated that the dramatic loss of these key proteins might lead to dendritic damage, and suggest general deficits across the brain.

The phosphorylation of MAP2 and stathmin can be activated by JNKs (Bjorkblom et al., 2005; Yip et al., 2014). JNKs, a subfamily of mitogen-activated protein kinases, are indispensable for the formation of the embryonic nervous system by regulating apoptotic and mitotic activities (Kuan et al., 1999; Waetzig and Herdegen, 2005). Recently, a novel mechanism for the regulation of brain development involved in MT stability by JNKs was found. Some studies have demonstrated that the growth of the functional dendritic architecture specifically depended on the cytoplasmic activity of JNK1, affluently localized in dendrites (Bjorkblom et al., 2005; Waetzig et al., 2006). In this study, we found that maternal exposure to DEHP can reduce expression of phospho-JNK1 in the hippocampus of male pups on PN7, PN14, and PN21. The phosphorylation of amino terminal residues in JNK can activate JNK to mediate downstream signaling transduction (Davis, 2000). In addition, studies have shown that JNK1^/-^ mice display a decrease in stathmin and MAP2 phosphorylation, as well as MT instability (Chang et al., 2003; Yip et al., 2014). The results suggest that the reason for perinatal exposure to DEHP leading to abnormal phosphorylation levels in MAP2 and stathmin, causing dendritic length and morphology abnormalities, may be attributed to the downregulation of active JNK1 in the hippocampus of male pups.

Our study suggested that maternal exposure to DEHP impaired the dendritic development in male offspring, but not in female offspring, and this may be attributed to the antiandrogen property of DEHP. Studies have shown that the levels and mRNA expression of proteins implicated in androgen synthesis were reduced following exposure to DEHP in early life (Gray et al., 2000; Parks et al., 2000; Borch et al., 2006; Lin et al., 2008). Furthermore, the low levels of prenatal androgens caused by the androgen receptor antagonist, flutamide, decreased the expression of MAP2 and induced reductions of dendritic branches in hippocampal neurons (Pallares et al., 2014). However, high-androgen pups exhibited hippocampal neurons with longer dendrites and a larger number of dendritic branches in early life and also performed better in a Morris water maze test (Isgor and Sengelaub, 2003).

Additionally, circulating androgens can be converted into estrogens in the brain via aromatase. Such a conversion is the primary source of estrogens in the male brain (Wu et al., 2009). Furthermore, locally synthesized estrogen in the brain can bolster estrogen effects on the increase in the dendritic length and the spine density of animals (Brocca et al., 2013). Studies have found that mono(2-ethylhexyl) phthalate, the active metabolite of DEHP, interferes with the metabolism of estrogens through the inhibition of aromatase enzyme activity (Hojo et al., 2008). Aromatase enzyme activity in the brain was attenuated on PN1 following perinatal exposure to low doses of DEHP in male offspring but not in females, while an increase in activity at weaning (PN22) was found in female but not in male pups (Andrade et al., 2006), further illustrating that DEHP exposure may decrease the level of estrogen in the brain of male pups. Thus, the above studies suggest that male animals are more susceptible to alteration of brain-derived estrogens caused by DEHP exposure. This may be one of the reasons why we observed the altered dendritic development and the dysregulation of related proteins in male offspring, while impairments in female offspring were not found.

## Conclusion

Our study showed that environmental exposure to DEHP during the critical period of brain development could decrease the total length and branching numbers of basal dendrites in the hippocampal CA1 subregion on PN7, PN14, and PN21. We also found that DEHP exposure may decrease the expression of MAP2c and stathmin predominantly regulating the synthesis of the neural cytoskeleton in a JNK1-dependent manner. Moreover, a negative impact on dendritic growth was prominent in males rather than females. This study indicated that DEHP exposure causes gender-specific damage to the dendritic growth of CA1 pyramidal neurons that may be related to the dysregulation of phosphorylated and total MAP2c and stathmin mediated by JNK1.

## Author Contributions

JC and JD conceived and directed the project. MY and JD planned the experiment in the whole study and wrote the manuscript. MY, YF, and ZC participated in the establishment of the animal model. MY, YF, ZC, and HF performed the experiments. YiW and YuW participated in the analysis of the experimental data. All authors contributed to revision of the manuscript.

## Conflict of Interest Statement

The authors declare that the research was conducted in the absence of any commercial or financial relationships that could be construed as a potential conflict of interest.

## Supplementary Material

FIGURE S1 Maternal exposure to DEHP does not cause significant changes to the bodyweight and the hippocampal weight of pups on PN7, PN14, and PN21. **(A,B)** Bar graphs display the results of bodyweight in male and female pups. **(C,D)** Bar graphs display the results of hippocampal weight in male and female pups.
